# Retraction Note: A water-soluble nucleolin aptamer-paclitaxel conjugate for tumor-specific targeting in ovarian cancer

**DOI:** 10.1038/s41467-026-71302-5

**Published:** 2026-03-27

**Authors:** Fangfei Li, Jun Lu, Jin Liu, Chao Liang, Maolin Wang, Luyao Wang, Defang Li, Houzong Yao, Qiulong Zhang, Jia Wen, Zong-Kang Zhang, Jie Li, Quanxia Lv, Xiaojuan He, Baosheng Guo, Daogang Guan, Yuanyuan Yu, Lei Dang, Xiaohao Wu, Yongshu Li, Guofen Chen, Feng Jiang, Shiguo Sun, Bao-Ting Zhang, Aiping Lu, Ge Zhang

**Affiliations:** 1 Institute of Precision Medicine and Innovative Drug Discovery (PMID), School of Chinese Medicine, Hong Kong Baptist University, Hong Kong SAR, 999077 China; 2 Institute for Advancing Translational Medicine in Bone and Joint Diseases (TMBJ), School of Chinese Medicine, Hong Kong Baptist University, Hong Kong SAR, 999077 China; 3 Institute of Integrated Bioinfomedicine and Translational Science (IBTS), School of Chinese Medicine, Hong Kong Baptist University, Hong Kong SAR, 999077 China; 4 College of Science, Northwest Agriculture and Forestry University, Yangling, Shaanxi China; 5 School of Chinese Medicine, Faculty of Medicine, The Chinese University of Hong Kong, Hong Kong SAR, 999077 China; 6 Department of Orthopaedics and Traumatology, Nanfang Hospital, Guangzhou, China

Retraction to: *Nature Communications* 10.1038/s41467-017-01565-6, published online 09 Novmber 2017

The Editors have retracted this article. After publication, concerns were raised regarding some of the data presented in Figs. 7 and 8, specifically:

• Fig. 7c CRO and NucA images appear to overlap.

• Fig. 7d PTX and NucA-PTX images appear to overlap.

• Fig. 8d Heart CRO and NucA-PTX images appear to overlap.

• Fig. 8d Liver PTX and NucA images appear to overlap.

• Fig. 8d Lung CRO and NucA images appear to overlap.

The authors have stated that a number of images in Figs. 7c, 7d, and 8d were misplaced due to labeling issues in the microscopy files, but have been unable to share the full underlying raw data upon request.

The Editors therefore no longer have confidence in the presented data.

Ge Zhang agrees with this retraction, but not the wording of this retraction notice. Shiguo Sun agrees with this retraction. The publisher has not been able to obtain a current email address for Jia Wen. The other authors have not responded to any correspondence from the editor or publisher about this retraction.

